# Effect of human mesenchymal stem cell secretome administration on morphine self-administration and relapse in two animal models of opioid dependence

**DOI:** 10.1038/s41398-022-02225-0

**Published:** 2022-11-04

**Authors:** María Elena Quintanilla, Mauricio Quezada, Paola Morales, Pablo Berríos-Cárcamo, Daniela Santapau, Marcelo Ezquer, Mario Herrera-Marschitz, Yedy Israel, Fernando Ezquer

**Affiliations:** 1 Molecular and Clinical Pharmacology Program, Institute of Biomedical Science, Faculty of Medicine, Universidad de Chile, Santiago, Chile; 2 Center for Regenerative Medicine, Faculty of Medicine Clínica Alemana-Universidad del Desarrollo, Santiago, Chile; 3 Department of Neuroscience, Faculty of Medicine, Universidad de Chile, Santiago, Chile; 4 Research Center for the Development of Novel Therapeutic Alternatives for Alcohol Use Disorders, Santiago, Chile

**Keywords:** Addiction, Stem cells

## Abstract

The present study investigates the possible therapeutic effects of human mesenchymal stem cell-derived secretome on morphine dependence and relapse. This was studied in a new model of chronic voluntary morphine intake in Wistar rats which shows classic signs of morphine intoxication and a severe naloxone-induced withdrawal syndrome. A single intranasal-systemic administration of MSCs secretome fully inhibited (>95%; *p* < 0.001) voluntary morphine intake and reduced the post-deprivation relapse intake by 50% (p < 0.02). Since several studies suggest a significant genetic contribution to the chronic use of many addictive drugs, the effect of MSCs secretome on morphine self-administration was further studied in rats bred as high alcohol consumers (UChB rats). Sub-chronic intraperitoneal administration of morphine before access to increasing concentrations of morphine solutions and water were available to the animals, led UChB rats to prefer ingesting morphine solutions over water, attaining levels of oral morphine intake in the range of those in the Wistar model. Intranasally administered MSCs secretome to UChB rats dose-dependently inhibited morphine self-administration by 72% (p < 0.001); while a single intranasal dose of MSC-secretome administered during a morphine deprivation period imposed on chronic morphine consumer UChB rats inhibited re-access morphine relapse intake by 80 to 85% (p < 0.0001). Both in the Wistar and the UChB rat models, MSCs-secretome administration reversed the morphine-induced increases in brain oxidative stress and neuroinflammation, considered as key engines perpetuating drug relapse. Overall, present preclinical studies suggest that products secreted by human mesenchymal stem cells may be of value in the treatment of opioid addiction.

## Introduction

Opioid dependence has become a public health emergency, with over 16 million opioid-dependent individuals worldwide [1, 2]. In 2022 the CDC’s National Center for Health Statistics reported an estimated 107,622 opioid overdose deaths in the United States during 2021, an increase of nearly 15% from the 93,655 deaths estimated in 2020. The situation is also dire in other countries; the World Health Organization (WHO 2021) [3] reports that over one half million deaths are attributable to drug use, and over 70% of these deaths are related to opioids. Thus, opioid addiction has become major cause of preventable morbidity and mortality worldwide. Recently, Harper et al. [4] informs that life expectancy in the United States had three consecutive years of decline. Among the causes for this decline the first one indicated is the increase in opiate overdose deaths. Noteworthy, a recent study further indicates that the increase in overdose mortality is explained largely by the availability and relatively low cost of opioids rather than by economic conditions [5].

Currently available treatments for opioid dependence mainly involve the administration of long-acting opioid agonists, such as methadone or buprenorphine, which have shown temporary efficacy in reducing illegal opioid use [6]. However, these substitution therapies maintain opioid dependence, leading to high rates of relapse upon treatment discontinuation [7]. Therefore, there is an urgent need to develop novel non-opioid-based pharmacological approaches.

Several preclinical and clinical studies have shown that brain oxidative stress and neuroinflammation are generated following the chronic administration of most drugs of abuse, including morphine, and are strongly associated with the perpetuation of drug intake and relapse [8–10]. Morphine consumption induces oxidative stress by the release of dopamine in the nucleus accumbens, which is oxidized by monoamine oxidase, generating hydrogen peroxide and hydroxyl radicals after its presynaptic re-uptake [8] and by the NO synthesis after μ-opioid receptor (MOR) activation [11]. Neuroinflammation also results from the chronic use of opioids [12], since morphine binds to the foreign-molecule sensor Toll-like receptor 4 (TLR4) in microglia and astrocytes, leading to the initiation of a signaling cascade that results in microglial and astrocytic activation and the production of pro-inflammatory mediators [10, 13, 14].

Morphine activation of MORs in the ventral tegmental area stimulates the reward system by increasing dopamine release into the nucleus accumbens and prefrontal cortex [15], playing a central role in the reinforcing properties of morphine [16]. Importantly, if after chronic use a prolonged drug deprivation is held, environmental cues associated with prior drug use trigger the urge to re-administer the drug, leading to relapse and the perpetuation of drug intake, a phenomenon that can persist for many years [16]. This behavior is driven by an elevation of extracellular glutamate in the tripartite synapse due to a cue-induced glutamate release into the nucleus accumbens [17–19].

Astrocytes surrounding the synapses are responsible for most of the glutamate re-uptake, playing a pivotal role in the termination of glutamatergic signaling [20]. However, this re-uptake is faulty in drug users as high levels of reactive oxygen species (ROS), by cross-linking of cysteine residues, can directly inhibit the main astrocyte glutamate transporter GLT-1 [21], and possibly the xCT cystine-glutamate antiporter [22], a carrier involved in glutamatergic downregulation. Neuroinflammation per se also lowers astrocyte GLT-1 levels and increases the extracellular glutamate levels [23]. Importantly, oxidative stress and neuroinflammation self-potentiate each other [24], being linked in *a long-acting vicious cycle* that is maintained in the brain of opioid users, altering glutamate homeostasis [8]. Therefore, interventions aimed at restoring glutamate homeostasis by the reduction of morphine-induced oxidative stress and neuroinflammation could help to reduce morphine dependence.

In recent years, mesenchymal stem cells (MSCs), an adult type of stem cell, have emerged as promising candidates for the treatment of neurological diseases commonly associated with neuroinflammation and oxidative stress [25, 26]. These cells have potent immunomodulatory properties, mainly mediated by the secretion of anti-inflammatory molecules which promote the inhibition of astrocyte and microglia activation [27], and by the production of high levels of antioxidant enzymes [28], which contribute to reduce oxidative stress. These anti-inflammatory and antioxidant properties are greatly enhanced by incubating MSCs with pro-inflammatory cytokines; a process known as preconditioning [29, 30].

Recently, it was shown that a single intra-cerebroventricular administration of rat MSCs [31] or human preconditioned MSCs [30] to rats that had consumed alcohol chronically reduced their voluntary ethanol intake by 75% and inhibited alcohol relapse upon re-access up to 85%. These effects were correlated with a significant reduction of neuroinflammation and oxidative stress and with an increase in GLT-1 levels in nucleus accumbens [30].

Initially MSCs were thought to exert their therapeutic effects by their multipotent differentiation capacity, replacing damaged cells in injured tissues. However, currently, the paracrine secretion (secretome) of a broad array of bioactive molecules is the most accepted therapeutic mechanism associated with MSCs [32, 33]. In line with this view, it was recently reported that the intranasal administration of cell-free secretome derived from preconditioned human MSCs to alcohol-dependent rats fully recapitulated the therapeutic effects of live MSC administration, including a 90% reduction in chronic alcohol intake, complete prevention of cue-induced alcohol relapse and fully reversion of hippocampal neuroinflammation and oxidative stress [34]. These effects were also shown after the intranasal administration of MSC-derived secretome in an animal model of nicotine dependence [34].

In the present study, using two rat models of oral morphine self-administration, we evaluated whether the administration of secretome derived from human preconditioned MSCs was able to reduce voluntary oral morphine intake and prevent relapse. The first model, as recently reported [10], required training animals to accept the bitter taste of morphine. For the second one, we used rats selectively bred to consume alcohol (UChB), which also have been shown to voluntarily consume nicotine [34, 35], in which voluntary oral morphine intake was induced after injecting priming doses of morphine. Further, we evaluated whether secretome administration was able to reverse molecular markers associated with opioid relapse, including morphine-induced neuroinflammation and oxidative stress and changes in opioid receptor and glutamate transporters mRNA levels. It is hypothesized that MSC-derived secretome administration will inhibit morphine self-administration and will markedly inhibit post-deprivation relapse.

## Materials and methods

### Animals

#### Model 1. Wistar rats

Just-weaned three-week-old female Wistar rats weighing 50–65 g were single-housed at constant temperature on a 12-h light/dark cycle and unrestricted access to standard rodent chow.

#### Model 2. Wistar-derived UChB rats

Eight-week-old female naïve rats weighing 95-120 g of the UChB line selectively bred for their high voluntary oral ethanol intake [36, 37] were single-housed at a constant temperature on a 12-h light/dark cycle with unrestricted access to rodent chow and water.

Both animal models were conducted with female rats since oral morphine consumption, or its self-administration is higher in females than in male rats [38–40]. Animal procedures were approved by the Committee for Experiments with Laboratory Animals of the Universidad del Desarrollo (Protocol-05/2020) and by the Committee for Experiments with Laboratory Animals of the University of Chile (Protocol-CBA# 0994-FMUCH).

### Voluntary morphine consumption and relapse

#### Animal model 1 (Wistar rats)

Voluntary oral consumption of morphine solution was induced in Wistar rats as recently described, where plasma morphine levels of 2.5 ± 0.6 μg/ml were attained after 4 weeks of voluntary intake [10]. Briefly, just-weaned rats were accustomed to the bitter taste of 150 mg/l quinine hydrochloride (Sigma-Aldrich) dissolved in tap water as their only fluid source available for seven days, after which animals were changed for two weeks to a two-bottle choice paradigm in which one bottle contained 150 mg/l quinine hydrochloride and the other 150 mg/l of the bitter morphine sulfate (Oramorph, Molteni Pharmaceutics) dissolved in tap water. Thereafter, animals were offered free-choice access between two bottles, one containing water and the other 150 mg/l morphine sulfate for two additional weeks. After 22 days of voluntary morphine intake animals were randomly divided into two groups that received a simultaneous intranasal (25μg protein) and intravenous (25 μg protein) dose of secretome derived from 1×10^6^ preconditioned MSCs (n = 9) or saline (n = 9) administered in a volume of 160μl. An additional group of animals drinking only water was used as control. To evaluate morphine post-deprivation relapse, in the same group of rats after 30 days of chronic morphine intake animals were morphine deprived for 12 days (water was available) and on days 2 and 9 of the deprivation period rats were treated with two simultaneous intranasal and intravenous administrations of secretome derived from preconditioned MSCs or vehicle. At the end of the deprivation period, animals were allowed morphine re-access for 24 hours to assess relapse, after which animals were euthanized (Supplementary Fig. 1A shows the time-course of experiment with Wistar rats).

#### Animal model 2 (UChB rats)

Voluntary oral morphine consumption was induced in UChB rats as previously described [41, 42] with some modifications. Briefly, rats were intraperitoneally administered a daily dose of 40 mg/kg of morphine hydrochloride (Sanderson Laboratory) in a volume of 2 ml/kg/day for 9 consecutive days. On day 10, after discontinuation of morphine injections, all rats were given free-choice access to two bottles: one containing water and the other morphine sulfate solution (Oramorph) of concentrations that were increased every two to four days, from 6 mg/l and reaching a steady intake of 50 mg/l on days 40 to 58. Thereafter, rats were given concurrently free choice access between three bottles: one with water and the other two containing 44 and 50 mg/l morphine sulfate from day 59 to 89. After 10 weeks of voluntary oral morphine consumption, animals were randomly divided into two groups (*n* = 4) that received an intranasal dose (25μg protein) of secretome derived from 1 × 10^6^ preconditioned MSCs or saline, administered in a total volume of 160μl/day, on days 75, 80 and 85. Thereafter, to evaluate morphine post-deprivation relapse, four days after the last intranasal secretome dose animals were deprived of the morphine solutions for five days and were administered a fourth dose of secretome or saline on the first day of the deprivation period. Subsequently, rats were allowed re-access to the 44- and 50 mg/l morphine solutions for one day.

To separately evaluate the effects of secretome on morphine relapse, secretome was intranasally administered only during the deprivation period to another group of sixteen UChB rats that were subjected to the same paradigm described for the first UChB group, but slightly modified to increase voluntary morphine intake and to induce a more potent morphine relapse. Rats were administered an intraperitoneal daily dose of 40 mg/kg of morphine hydrochloride in a volume of 2 ml/kg/day for 11 consecutive days. After discontinuation of morphine injections on day 12, rats were offered a free-choice between a morphine solution of increasing concentration on successive days (6 to 90 mg/l morphine) or water until day 42. Thereafter, rats were given concurrently free-choice access between three bottles: one containing water and the other two containing 80 or 90 mg/l morphine sulfate solutions from day 43 to 57. After eight weeks of continuous voluntary morphine consumption, animals were deprived of morphine for six days and on the fourth day of morphine deprivation, animals were randomly divided into 2 groups (n = 6) and were treated with a single intranasal dose of secretome (25μg protein) derived from 1×10^6^ preconditioned MSCs or saline administered in a volume of 160μl. Rats continued under morphine deprivation for two additional days and thereafter animals were allowed re-access to the 80- and 90 mg/l morphine solutions for two days (Supplementary Fig. 1B and C show the time-course of experiments with UChB rats).

In both animal models, the bottle positions were alternated every day to avoid the development of a side preference. Morphine intake, water intake, and animal body weight were measured daily. In both models reported, morphine is mainly consumed by the animals in the dark period of the circadian cycle.

### Isolation, expansion and characterization of human adipose tissue-derived MSCs

Human MSCs were isolated from fresh subcutaneous adipose tissue samples obtained from liposuction aspirates of patients undergoing cosmetic liposuction at Clínica Alemana, Santiago, Chile after obtaining written informed consent as previously reported [34, 43]. Protocols were approved by the Ethics Committee of Faculty of Medicine, Clínica Alemana-Universidad del Desarrollo. After two subcultures, cells were characterized following the minimum criteria established by the International Society for Cellular Therapy [44] as previously described [34, 43].

### Preconditioning of MSCs and secretome generation

MSCs (passage 3) at 75% confluency were preconditioned to improve the production of anti-inflammatory and antioxidant molecules by incubation in minimal essential medium (α-MEM, Gibco) supplemented with 10% fetal bovine serum (FBS, Hyclone) plus 10 ng/ml TNF-α (R&D System) and 15 ng/ml IFN-γ (R&D System) for 40 hours as previously described [30, 34, 45]. After preconditioning, cells were washed and incubated for 48 hours in α-MEM without FBS. Then, culture medium (secretome) was collected and centrifuged at 400 g for 10 minutes to remove detached cells. The supernatant was centrifuged again at 5,000 g for 10 minutes to remove cell debris. Finally, secretome was filtered with 0.22 μm filters and concentrated 50 times (v/v) using 3 kDa cutoff filters (Millipore). Protein concentration was determined by the BCA protein assay kit (Thermo Scientific) and secretome was aliquoted and frozen at −80 °C until use.

### Non-invasive administration of secretome derived from human preconditioned MSCs

A. ***Intranasal administration*:** Rats were anesthetized by intramuscular administration of ketamine (60 mg/kg) and acepromazine (4 mg/kg) [46] and kept in supine position. Twenty microliters of secretome solution were administered intranasally as small drops delivered from a pipette tip every five minutes into alternative sides of the nasal cavity (four times in each nostril) for a total of 40 minutes. A total volume of 160μl of secretome containing 25μg of proteins derived from 1 × 10^6^ preconditioned MSCs was delivered into the nasal cavity. Vehicle-treated animals received 160μl of saline solution by the same administration scheme as previously reported [34].
B. ***Intravenous administration:*** In the Wistar rat (Animal Model #1) studies, in addition to the intranasal administration animals received secretome by the intravenous route. Immediately after the intranasal administration, animals were injected via the tail vein with 160μl of secretome containing 25μg of proteins derived from 1 × 10^6^ preconditioned MSCs. Vehicle-treated animals received 160μl of saline solution using the same administration scheme.

### Evaluation of morphine-induced neuroinflammation

In both animal models, neuroinflammation was evaluated as previously described [34, 47], determining astrocyte activation and microglial density in the hippocampus of rats after the 24-hours morphine re-access. Briefly, after the last morphine intake register animals were anesthetized by the intramuscular administration of 1.9 ml/kg of an anesthetic cocktail (70 mg/kg ketamine, 10 mg/kg xylazine and 4 mg/kg acepromazine) [48], intracardially perfused with 0.1 M PBS (pH 7.4) and euthanized. Double-labeling immunofluorescence against the astrocyte marker glial fibrillary acidic protein (GFAP) (Sigma-Aldrich G3893, 1:500 dilution) and the microglial marker ionized-calcium-binding adaptor molecule 1 (Iba-1) (Wako 019-19741, 1:400 dilution) were performed in coronal 30 μm thick cryo-sections of hippocampus. Nuclei were labeled with DAPI (Invitrogen). Microphotographs were taken from the *stratum radiatum* of hippocampus using a confocal microscope (Zeiss, LMS700). The area analyzed for each stack was 0.04mm^2^. The total length and thickness of primary astrocytic processes and the density of microglial cells were determined using FIJI image analysis software as previously reported [34, 47].

### Evaluation of morphine-induced oxidative stress

In both animal models, after the 24-hour morphine re-access brain oxidative stress was evaluated determining the ratio between oxidized glutathione/reduced glutathione (GSSG/GSH) in the hippocampus and the levels of lipid peroxidation in the neostriatum. The GSSG/GSH ratio was determined as we previously reported [34, 47]. Lipid peroxidation was determined by a method that measures the amount of malondialdehyde (MDA) formed using the Lipid Peroxidation assay kit (Sigma-Aldrich) as we previously described [49].

### Determination of mRNA levels of opioid receptors and glutamate transporters

After the 24-hour morphine re-access, total RNA from prefrontal cortex and nucleus accumbens was purified using TRIzol reagent (Invitrogen). One microgram of RNA was used to perform reverse transcription with MMLV reverse transcriptase (Invitrogen) and oligo dT primers. Real-time PCR reactions were performed to amplify the μ-opioid receptor 1 (OPRM1) and the glutamate transporters GLT-1 and xCT, using specific primers (Supplementary Table 1) and SYBRGreen reagent (Roche) in a LightCycler thermocycler (Roche). Relative quantifications were performed by the ΔΔCT method. The mRNA level of each target gene was normalized against the mRNA level of the housekeeping gene glyceraldehyde-3-phosphate dehydrogenase (GAPDH). All biochemical analysis were performed in a single-blind way.

### Statistical analysis

Data are expressed as means ± SEM. Statistical analyses were performed using GraphPad Prism v.9.2.0 software. The normal distribution of data for all experiments was shown using the Shapiro-Wilk test. Bartlett’s test showed that the variances between groups were equal. Two-way (treatment x day) ANOVA, followed by Tukey’s post-hoc test or One-way ANOVA, followed by Tukey’s post-hoc test was conducted to compare differences between experimental groups. Two-tailed Student t test was performed to determine if there is statistical significance when only two groups were compared. A level of p < 0.05 was considered for statistical significance.

## Results

### Model 1: Wistar rats

#### Simultaneous intranasal and intravenous administration of secretome derived from human preconditioned MSCs to Wistar rats inhibits chronic morphine intake and reduces post-deprivation morphine re-access intake

**Fig. 1:**
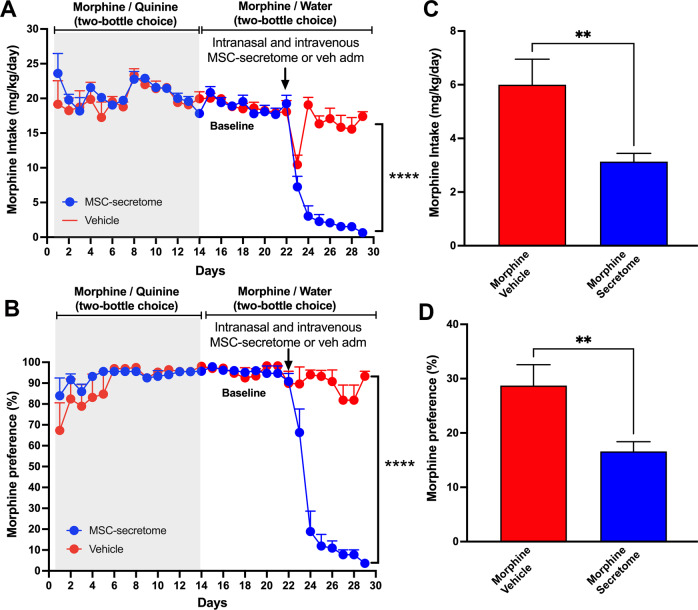
Simultaneous intranasal and intravenous administration of secretome derived from human preconditioned MSCs to Wistar rats that had chronically consumed morphine inhibits morphine intake and reduces post-deprivation morphine re-access intake. **A** Daily morphine intake and (**B**) morphine preference over water. Wistar rats that had voluntarily ingested morphine for 21 days were simultaneously intranasally and intravenously treated with secretome (25μg protein) derived from 1 × 10^6^ preconditioned MSCs or vehicle (indicated by the arrow). Morphine intake was inhibited by 95% (*p* < 0.0001). Two-way ANOVA (treatment x day) of morphine intake and morphine preference following secretome administration (blue circles) indicate significant effect of treatment (F_*(1,435)*_ = 158.9 ****p < 0.0001), day (F_*(28,435)*_ = 30.4, *p* < 0.0001) and significant interaction (F treatment x day _*(28,435)*_ = 25.2, *p* < 0.0001) compared with control animals receiving vehicle (red circles). **C** Morphine relapse intake and (**D**) morphine preference over water after 12 days of morphine deprivation followed by 24-hours of morphine re-access. Rats treated previously with three intranasal and intravenous secretome doses (on day 22 of chronic morphine access and on days 2 and 9 of the deprivation period) (blue bars) ingested significantly lower (−50%) amount of morphine compared with vehicle-treated animals (red bar) (**p < 0.01, two-tailed Student t test). Data are presented as mean ± SEM; n = 9 for each experimental condition.

In this model of opioid dependence [10], just-weaned Wistar rats were exposed for seven days to a quinine solution as their only fluid source to get the animals accustomed to a bitter taste (Fig. 1 day zero), after which animals were offered a two-bottle choice of quinine or morphine solutions, for two weeks. Figure 1A (shaded area) shows that Wistar rats that could choose between a 150 mg/l quinine solution versus one of 150 mg/l morphine achieved a high morphine consumption (20.1 ± 0.4 mg/kg/day) for 14 days. Four animals (15%) kept a significant quinine preference (below 50% preference for the morphine solution) and were removed from the study. Subsequently, animals were offered a two-bottle choice between a morphine solution (150 mg/l) and water from day 15 onwards, showing a voluntary morphine intake of 18.5 ± 0.3 mg/kg/day (Fig. 1A), with a preference for morphine solution over water above 85% (Fig. 1B). As previously reported [10], these levels of morphine intake allow the development of strong naloxone-induced withdrawal signs of dependence. In the present study, five animals changed their preference ratio from the morphine solution to water and were removed from the study. Thus, the overall rejection corresponds to 9 animals of the original 27 animals (33%), in line with a previous report [10]. After removing the animals that changed morphine preference, the remaining animals were distributed into two groups according to their morphine intake on the last three days, such as to avoid group differences. Prior to initiating the secretome or vehicle administration, the average 3-day intake was 18.1 ± 0.7 mg/kg/day for the animals in the vehicle treated group and 18.3 ± 0.7 mg/kg/day for the animals in the secretome treated group.

To evaluate the therapeutic potential of MSCs, the secretome derived from 1 × 10^6^ preconditioned human adipose tissue-derived MSCs (25μg protein) was administered simultaneously intranasally and intravenously to one half of the rats that had voluntarily consumed morphine for 22 days (n = 9), while the other half were given saline (vehicle) as control (n = 9). Secretome administration virtually abolished morphine dependence (p < 0.0001, Two-way ANOVA) (Fig. 1A), reaching >95% reduction in morphine intake seven days after the single-time secretome administration compared with vehicle-treated animals (0.6 ± 0.3 mg morphine consumed/kg/day for secretome-treated animals versus 17.4 ± 0.7 mg morphine consumed/kg/day for vehicle-treated animals). Noteworthy, most of the inhibitory effect on morphine intake was seen within the first 48 hours (>80% reduction in morphine intake). Secretome administration also reduced morphine preference versus water (p < 0.0001, Two-way ANOVA) (Fig. 1B). The reduction in morphine intake induced by secretome administration was fully compensated by an increase in water intake (Supplementary Fig. 2A); additionally, secretome administration did not affect animal body weight (Supplementary Fig. 2B), both suggesting that the effects induced by this treatment were specific for morphine intake and do not reflect a nonspecific malaise nor severe withdrawal symptoms.

To evaluate the effect of secretome administration on post-deprivation relapse intake after morphine re-access, seven days after the administration of secretome or vehicle animals were morphine-deprived for 12 days and treated with two intranasal and intravenous administrations of secretome or vehicle (on days 2 and 9 of the deprivation period) and allowed morphine (150 mg/l) re-access for 24 hours (water was always available through both the deprivation period and morphine re-access). We observed that upon morphine re-access, secretome-treated animals ingested a significantly lower amount of morphine (p < 0.01, Two-tailed Student t test) (Fig. 1C) and showed a reduced morphine preference (p < 0.01, Two-tailed Student t test) (Fig. 1D) compared with vehicle-treated animals.

#### Simultaneous intranasal and intravenous administration of secretome derived from human preconditioned MSCs to Wistar rats chronically ingesting morphine normalizes the increased astrocyte reactivity and brain oxidative stress induced by morphine intake

**Fig. 2:**
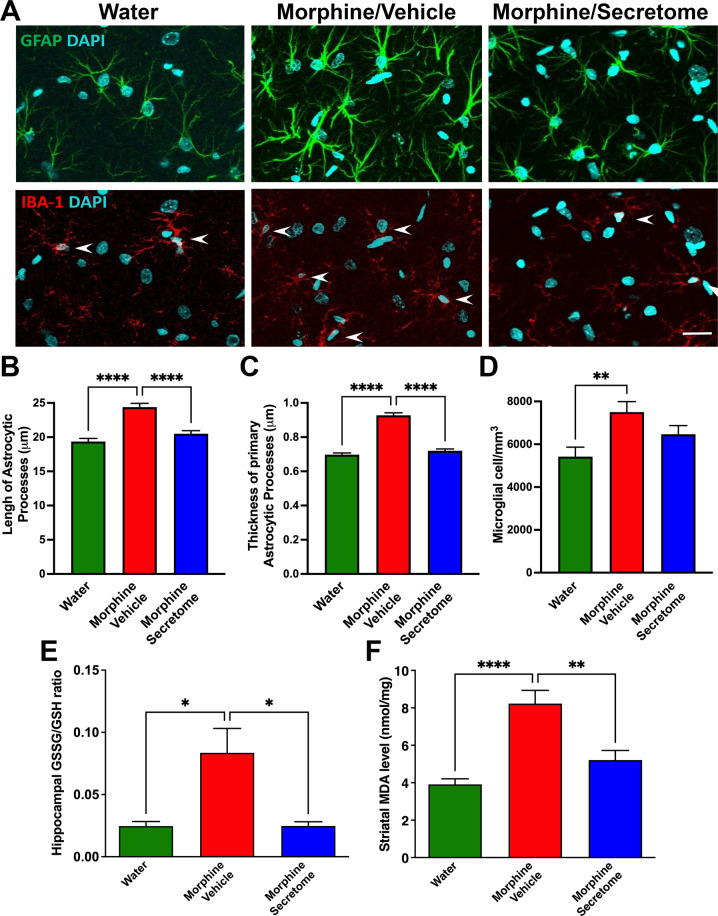
Simultaneous intranasal and intravenous administration of secretome derived from human preconditioned MSCs normalizes the increased astrocyte reactivity and brain oxidative stress induced by chronic morphine intake in Wistar rats. **A** Representative confocal microphotographs of GFAP immunoreactivity (green top) in hippocampal astrocytes and Iba-1 immunoreactivity (red, shown by arrows, center) in hippocampal microglia. Nuclei were counterstained with DAPI (blue, nuclear marker), scale bar 25 μm. **B** Quantification of length and (**C**) thickness of primary astrocytic processes. **(D)** Quantification of microglial density. Chronic morphine-drinking rats treated with vehicle displayed a marked increase in the length and thickness of astrocytic processes (**** *p* < 0.0001, One-way ANOVA followed by Tukey’s post-hoc test) and microglial density (red bars in **B**–**D**) (**p < 0.01, One-way ANOVA followed by Tukey’s post-hoc test) compared with water drinking rats (green bars in **B**–**D**). The simultaneous intranasal and intravenous administration of three secretome doses (on day 22 of the chronic morphine access and on days 2 and 9 of the deprivation period) significantly reduced the length and thickness of primary astrocytic processes (blue bars in **B** and **C**) (****p < 0.0001, One-way ANOVA followed by Tukey’s post-hoc test), but not the microglial density (blue bar in **D**) compared with the morphine + vehicle treated animals (red bars in **B**–**D**). (E) GSSG/GSH ratio in the hippocampus of rats chronically drinking morphine, deprived for 12 days, and allowed morphine re-access for 24 hours. Chronic morphine drinking rats treated with vehicle showed a three-fold increase in the GSSG/GSH ratio (red bar) (*p < 0.05, One-way ANOVA followed by Tukey’s post-hoc test) compared with rats drinking only water (green bar). The simultaneous intranasal and intravenous administration of three secretome doses (on day 22 of the chronic morphine access and on days 2 and 9 of the deprivation period) resulted in the full normalization of the GSSG/GSH ratio (blue bar) (*p < 0.05, One-way ANOVA followed by Tukey’s post-hoc test). **F** MDA levels in the neostriatum. Chronic morphine drinking rats treated with vehicle showed a two-fold increase in MDA levels (red bar) (****p < 0.0001, One-way ANOVA followed by Tukey’s post-hoc test) compared with rats drinking only water (green bar). Simultaneous intranasal and intravenous administration of three secretome doses resulted in the near complete normalization of the MDA levels (blue bar) (**p < 0.01, One-way ANOVA followed by Tukey’s post-hoc test). Data are presented as mean ± SEM; n = 9 for each experimental condition.

Glial cells are the central players in the induction and maintenance of brain inflammation [50]. As indicated, neuroinflammation and oxidative stress self-perpetuate each other and may be responsible for relapse drug intake despite prolonged drug abstinence [8]. Thus, neuroinflammation was evaluated determining glial reactivity, evidenced as morphological changes in astrocytes and microglial density in the hippocampus. To determine whether secretome administration reduced morphine-induced neuroinflammation animals were euthanized immediately after the 24-hour morphine re-access and their hippocampi were processed for astrocyte and microglia immunofluorescence. Animals that were never exposed to morphine (water group) were used as controls. As expected, chronic morphine intake induced a marked astrocytosis evidenced by a significant increase in the length (p < 0.0001, One-way ANOVA followed by Tukey’s post-hoc test) (Fig. 2A top and B) and thickness (p < 0.0001, One-way ANOVA followed by Tukey’s post-hoc test) (Fig. 2A top and C) of the primary astrocytic processes compared with those of water drinking animals. Secretome administration fully reversed the increase in the length and thickness of primary astrocytic processes (p < 0.0001, One-way ANOVA followed by Tukey’s post-hoc test) (Fig. 2A top and B and C). Morphine ingesting animals also showed a significant increase in microglial density (p < 0.01, One-way ANOVA followed by Tukey’s post-hoc test) (Fig. 2A center and D) compared with those of water drinking animals. Secretome administration had no significant impact on morphine-increased microglial density (Fig. 2A center and D).

Oxidative stress was determined as the ratio of oxidized/reduced glutathione (GSSG/GSH) in the hippocampus and by the level of lipid peroxidation in the neostriatum (two brain structures of the reward system) since both are highly sensitive hallmark indicators of the brain cellular redox state [51]. We observed that GSSG/GSH ratio in the hippocampus of rats chronically ingesting morphine, deprived of morphine for 12 days and allowed morphine re-access for 24 hours was increased 3.5-fold compared to that in water drinking animals (p < 0.05, One-way ANOVA followed by Tukey’s post-hoc test) (Fig. 2E). Three intranasal and intravenous administrations of MSC-derived secretome (one during the chronic morphine intake and two during the deprivation period) fully restored the normal GSSG/GSH ratio (p < 0.05, One-way ANOVA followed by Tukey’s post-hoc test) (Fig. 2E). Similar results were observed for the evaluation of MDA levels in the neostriatum (Fig. 2F); thus, indicating that protracted neuroinflammation and oxidative stress induced by voluntary morphine intake were suppressed by secretome administration.

#### Simultaneous intranasal and intravenous administration of secretome derived from human preconditioned MSCs to Wistar rats chronically ingesting morphine does not modify the increase of morphine-induced expression of neither μ-opioid receptor nor glutamate transporters

**Fig. 3:**
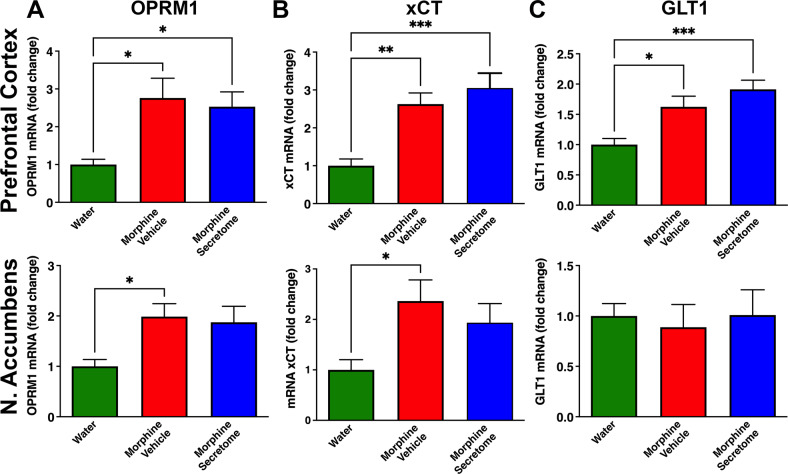
Simultaneous intranasal and intravenous administration of secretome derived from human preconditioned MSCs to Wistar did not modify the morphine-induced increases in the brain expression of μ-opioid receptor or glutamate transporters. Quantification of the mRNA levels of the (**A**) μ-opioid receptor OPRM1; (**B**) xCT glutamate transporter and (**C**) GLT-1 glutamate transporter, determined by RT-qPCR in prefrontal cortex (top) and nucleus accumbens (bottom) of rats chronically drinking morphine, deprived for morphine for 12 days and allowed morphine re-access for 24 hours. Data were normalized against the mRNA level of the housekeeping gene GAPDH in the same sample. Morphine intake induced a significant increase in μ-opioid receptor mRNA levels in prefrontal cortex and nucleus accumbens (red bars in **A**) (*p < 0.05, One-way ANOVA followed by Tukey’s post-hoc test), in xCT mRNA levels in prefrontal cortex and nucleus accumbens (red bars in **B**) (*p < 0.05, **p < 0.01 One-way ANOVA followed by Tukey’s post-hoc test), and in GLT-1 mRNA levels in prefrontal cortex (red bars in **C**) (*p < 0.05, One-way ANOVA followed by Tukey’s post-hoc test), compared with water drinking animals (green bars). Simultaneous intranasal and intravenous administration of three secretome doses (on day 22 of the chronic morphine access and on days 2 and 9 of the deprivation period) had no impact on the mRNA levels of these molecules. Data are presented as mean ± SEM; n = 9 for each experimental condition.

The μ-opioid receptor 1 (OPRM1) is the main subtype receptor for morphine in the central nervous system [52], exhibiting a clear role in morphine-induced analgesia and morphine rewarding effects and dependence [53]. Changes in the activity of glial glutamate transporters, which regulate glutamate uptake have also been implicated in the development of morphine dependence [54]. Thus, we measured in prefrontal cortex and nucleus accumbens the mRNA levels of (a) OPRM1, (b) xCT and (c) GLT-1, 24 hour after morphine re-access. Morphine drinking animals showed a significant increase in mRNA levels of OPRM1 (p < 0.05, One-way ANOVA followed by Tukey’s post-hoc test) in prefrontal cortex and nucleus accumbens (Fig. 3A) and, an unexpected increase in xCT mRNA level (p < 0.05, One-way ANOVA followed by Tukey’s post-hoc test) in prefrontal cortex and nucleus accumbens (Fig. 3B) and in GLT-1 mRNA level (p < 0.05, One-way ANOVA followed by Tukey’s post-hoc test) in prefrontal cortex only (Fig. 3C) compared to water drinking animals. Secretome administration to morphine dependent animals did not alter morphine-induced mRNA levels of OPRM1, xCT and GLT-1 since differences in the mRNA levels of these molecules were not significant compared to morphine dependent animals treated with the vehicle.

### Model 2: Wistar-derived UChB rats

#### Intranasal administration of secretome derived from human preconditioned MSCs inhibits chronic morphine intake and reduces post-deprivation morphine re-access intake in UChB rats

**Fig. 4:**
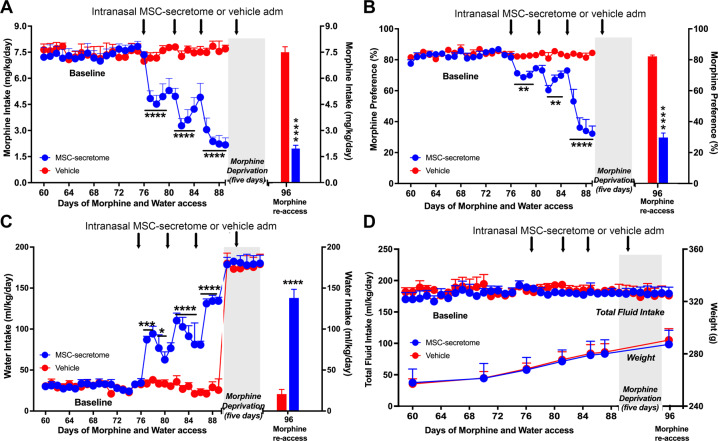
Repeated intranasal administration of secretome derived from human preconditioned MSCs to UChB rats that had chronically consumed morphine inhibits its intake and reduces post-deprivation relapse. **A**-Left Daily morphine intake and (**B**-Left) morphine preference over water. UChB rats that had consumed morphine for 76 days were intranasally treated with three doses of secretome derived from preconditioned MSCs or vehicle (indicated by arrows). Two-way ANOVA (treatment × day) of morphine intake following three intranasal doses of MSC secretome indicates significant effect of treatment [*F*_*(1,180)*_ = 195.4, *p* < 0.0001], day [*F*_*(29,180)*_ = 8.76, *p* < 0.0001], and significant interaction [*F*_*(29,180)*_ = 10.15, *p* < 0.001] compared with control rats receiving vehicle. Tukey´s post-hoc analysis revealed that secretome treatment (blue circles) inhibited morphine intake during the 14 days recorded period, versus vehicle-treated control (red circles) (***p < 0.001). The inhibition of morphine intake induced by the third secretome dose (72%) was significantly higher (**p < 0.01) than the inhibition induced by the second dose (50%) and the inhibition induced by the latter was significantly higher (*p < 0.05) than the inhibition induced by the first dose (34%) (two-tailed Student t test). (**A** Right) Morphine intake and (**B** Right) morphine preference over water of UChB rats after five days of morphine deprivation followed by one day of free choice of 44 mg/l and 50 mg/l morphine re-access. Rats treated previously with four intranasal secretome doses (blue bar) ingested a 74% lower amount of morphine than vehicle treated animals (red bar) (****p < 0.0001; Two-tailed Student t test). (**C** Left) Daily water intake showing that intranasal administration of three secretome doses (blue circles) significantly increased water intake compared with vehicle treated rats (red circles). Two-way ANOVA (treatment × day) indicates significant effect of treatment [*F*_*(1,216)*_ = 213.9, *p* < 0.0001], day [*F*_*(35,216)*_ = 104.7, *p* < 0.0001], and significant interaction [*F*_*(35, 216)*_ = 11.92, *p* < 0.0001]. Tukey’s post-hoc analysis revealed that secretome treatment increases water intake during the 14 days recorded versus vehicle-treated control (***p < 0.001). The higher water intake induced by the third secretome dose (80%) was significantly higher than the increase induced by the first dose (57%) (*p < 0.05, two-tailed Student t test). (**C** Right) Water intake after five days of morphine deprivation followed by one day of 44 mg/l and 50 mg/l morphine solutions re-access. Rats treated previously with four intranasal secretome doses (blue bar) ingested a significantly higher amount of water than vehicle control animals (red bar) (****p < 0.0001, Two-tailed Student t test). (**D**) Total fluid intake and animals body weights of UChB rats intranasally treated with secretome derived from preconditioned MSCs (blue circles) or vehicle (red circles). Data showed that body weight or total fluid intake were not affected by secretome administration, indicating that effects induced by this treatment were specific for morphine intake. Data are presented as mean ± SEM; n = 4 for each experimental condition.

Numerous studies have identified the contribution of heredity on drug dependence and the possible role of common pathways associated with dependence for multiple drugs [55]. The Wistar-derived UChB rat line, selectively bred during ninety generations for its high voluntary ethanol consumption [36, 37], appears as a useful model to study oral poly-drug addiction [34, 35]. Thus, we evaluated if secretome administration could reduce chronic morphine intake in the UChB rat model. UChB rats were pretreated with a daily intraperitoneal dose of morphine (40 mg/kg) for 9 days to promote subsequent voluntary morphine intake. After discontinuation of systemic morphine administration, the animals were offered free-choice access between two bottles, one containing water and the other a morphine solution of increasing concentrations on successive days; a paradigm in line with studies of nicotine self-administration [41, 42] (Supplementary Fig. 1B). A progressive escalation of voluntary oral morphine consumption was achieved by increasing the concentration of the morphine solution offered (Supplementary Fig. 3A). Following 15 days of access to 44 and 50 mg/l morphine solutions and water, animals achieved a stable oral morphine consumption of 7.4 ± 0.2 mg morphine/kg/day. After 10 weeks of continuous voluntary morphine consumption, animals were divided into two groups and treated with an intranasal dose of secretome (25μg protein) derived from 1 × 10^6^ preconditioned MSCs or vehicle. It has been previously reported that, by this route of administration, MSC-secretome is effective in reducing voluntary alcohol and nicotine intake in UChB rats [34]. As shown in Fig. 4A**-**Left, the first intranasal secretome administration induced a 37% reduction (p < 0.0001, Two-way ANOVA) in voluntary morphine intake compared to that of vehicle-treated animals. The intranasal administration of additional secretome doses given every five days for two weeks significantly enhanced (p < 0.0001, Two-tailed Student t test) the inhibitory effect of secretome, reaching a 72% reduction in morphine intake after three intranasal secretome doses (2.2 ± 0.4 mg morphine/kg/day for secretome-treated animals versus 7.7 ± 0.2 mg morphine/kg/day for vehicle-treated animals). The half-life of the secretome inhibitory action on morphine intake is of the order of 6-7 days, such that the secretome administration every 5 days results in a cumulative effect. As expected, the reduction in morphine intake induced by secretome administration was associated with a significant reduction (p < 0.01 to 0.0001, Two-way ANOVA) in morphine preference versus water (Fig. 4B**-**Left). Secretome-induced inhibition of morphine intake was counterbalanced by increases in water intake (p < 0.0001, Two-way ANOVA) (Fig. 4C Left), thus indicating that rats kept their hydric homeostasis. Four days after the last intranasal dose of secretome, animals were deprived of the morphine solutions for five days and treated with a fourth dose of secretome or vehicle during the first day of deprivation. Subsequently, rats were allowed re-access to the 44- and 50 mg/l morphine solutions for one day to evaluate post-deprivation relapse intake. Animals in the vehicle-treated group ingested 7.2 ± 0.2 mg morphine/kg/day while animals in the secretome-treated group ingested 2.0 ± 0.2 mg morphine/kg/day; thus, a 72% reduction (p < 0.0001, Two-tailed Student t test) in post-deprivation morphine relapse intake (Fig. 4A**-**Right). Secretome administration also significantly reduced (p < 0.0001, Two-tailed Student t test) morphine preference over water upon re-access (Fig. 4B**-**Right). As also observed in the Wistar rat model, secretome administrations had no impact on total fluid intake or animal weight in UChB rats (Fig. 4D).

#### Intranasal administration of secretome derived from human preconditioned MSCs reduces morphine-induced astrocyte activation and brain oxidative stress in UChB rats

**Fig. 5:**
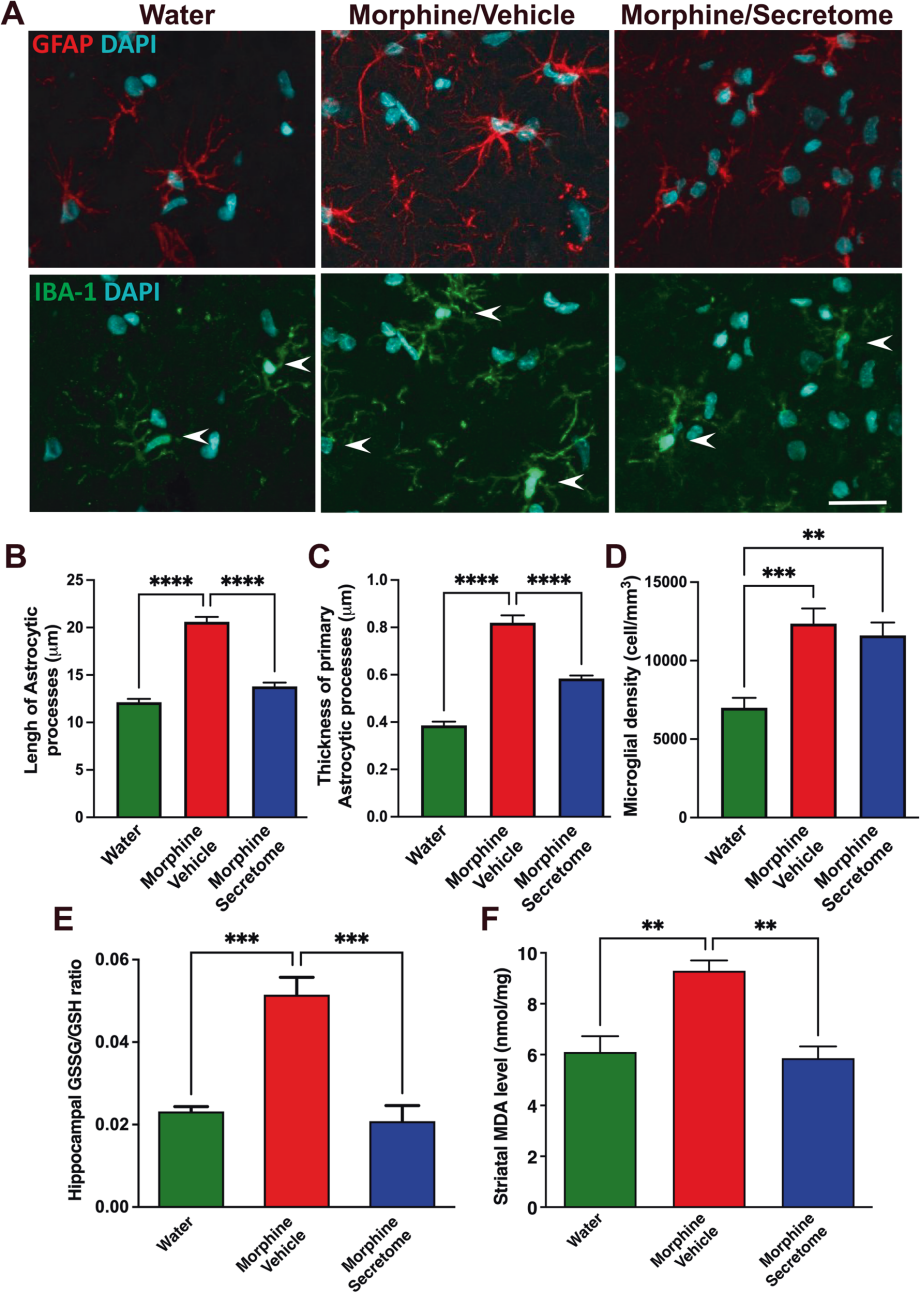
Intranasal administration of secretome derived from human preconditioned MSCs reduces morphine-induced astrocyte activation and brain oxidative stress in UChB rats. **A** Representative confocal microphotographs of GFAP immunoreactivity (red top) for hippocampal astrocytes and Iba-1 immunoreactivity (green, shown by arrows, center) for hippocampal microglia. Nuclei were counterstained with DAPI (blue, nuclear marker), scale bar 25 μm. **B** Quantification of length and (**C**) thickness of primary astrocytic processes. **D** Quantification of microglia density. Chronic morphine-ingesting rats treated with vehicle displayed a marked increase in the length and thickness of astrocytic processes (****p < 0.0001, One-way ANOVA followed by Tukey’s post-hoc test) and microglial density (red bars in **B**–**D**) (***p < 0.001, One-way ANOVA followed by Tukey’s post-hoc test) compared with water drinking rats (green bars in **B**–**D**). The intranasal administration of secretome significantly reduced the length and thickness of primary astrocytic processes (blue bars in **B** and **C**) (****p < 0.0001, One-way ANOVA followed by Tukey’s post-hoc test) compared with the morphine vehicle treated animals (red bars in **B** and **C**). **E** Quantification of GSSG/GSH ratio in the hippocampus. Chronic morphine drinking rats treated with vehicle showed a 2.5-fold increase in GSSG/GSH ratio (red bar) (***p < 0.001, One-way ANOVA followed by Tukey’s post-hoc test) compared with rats drinking only water (green bar). Intranasal administration of secretome resulted in the fully normalization of the GSSG/GSH ratio (blue bar) (***p < 0.001, One-way ANOVA followed by Tukey’s post-hoc test). **F** Quantification of MDA levels in neostriatum. Chronic morphine drinking rats treated with vehicle showed a 60% increase in MDA levels (red bar) (**p < 0.01, One-way ANOVA followed by Tukey’s post-hoc test) compared with rats drinking only water (green bar). Intranasal secretome administration resulted in the full normalization of MDA levels (blue bar) (**p < 0.01, One-way ANOVA followed by Tukey’s post-hoc test). Data are presented as mean ± SEM; n = 4 for each experimental condition.

As for the Wistar rat model, we evaluated whether chronic morphine intake also induced neuroinflammation and brain oxidative stress in UChB rats and whether these parameters could be normalized by secretome administration. To this end, animals were euthanized after the 24-hour morphine re-access. Animals that were never exposed to morphine (water group) were used as controls. Once again, chronic morphine intake induced a marked astrocytosis evidenced by a significant increase in the length (p < 0.0001, One-way ANOVA followed by Tukey’s post-hoc test) (Fig. 5A-top and Fig. 5B) and thickness (p < 0.0001, One-way ANOVA followed by Tukey’s post-hoc test) (Fig. 5A-top and C) of primary astrocytic processes in hippocampus compared with those in water drinking animals. Secretome administration fully reversed the increase in the length and thickness of primary astrocytic processes (p < 0.0001, One-way ANOVA followed by Tukey’s post-hoc test) (Fig. 5A-top, B and C). Morphine ingesting animals also showed a significant increase in microglial density (p < 0.001, One-way ANOVA followed by Tukey’s post-hoc test) (Fig. 5A-center and D) compared with that in water drinking animals, while the administration of secretome had no impact on morphine-increased microglial density (Fig. 5A-center and D).

As also seen in Wistar rats, UChB rats showed a significant increase (p < 0.001, One-way ANOVA followed by Tukey’s post-hoc test) of GSSG/GSH ratio in hippocampus (Fig. 5E) and of MDA levels (p < 0.01, One-way ANOVA followed by Tukey’s post-hoc test) in neostriatum **(**Fig. 5F**)** of morphine-drinking compared to water drinking animals. Intranasal secretome administration fully restored the GSSG/GSH ratio (p < 0.001, One-way ANOVA followed by Tukey’s post-hoc test) (Fig. 5E) and the MDA levels (p < 0.01, One-way ANOVA followed by Tukey’s post-hoc test) to levels observed in animals that were not exposed to morphine (Fig. 5F).

#### Single intranasal administration of secretome derived from human preconditioned MSCs during the morphine deprivation period reduces morphine relapse in UChB rats

Subsequent studies evaluated whether the administration of a *single intranasal dose* of secretome derived from preconditioned MSCs, given only during the deprivation period, reduced the post-deprivation morphine relapse upon morphine re-access. To increase voluntary morphine intake and to induce a more potent morphine relapse, the previous paradigm of morphine self-administration was slightly modified. The priming schedule of daily intraperitoneal administration of 40 mg/kg morphine was extended from 9 to 11 days and the morphine concentrations offered on the subsequent two-bottle choice was increased at a faster pace, reaching 90 mg/l within 41 days of morphine exposure (Supplementary Fig. 1C). Rats subjected to this paradigm consumed 14.1 ± 0.1 mg of morphine/kg/day (Supplementary Fig. 3B).

**Fig. 6:**
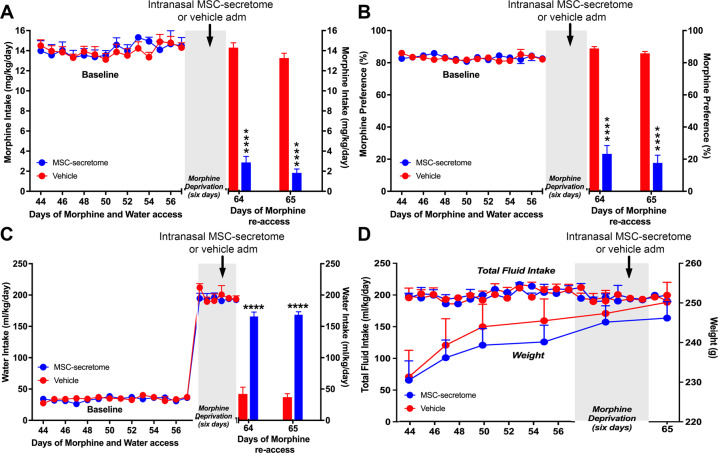
A single intranasal administration of secretome derived from human preconditioned MSCs during the deprivation period to UChB rats that had chronically consumed morphine markedly reduces post-deprivation relapse. **A** Morphine relapse intake and (**B**) Morphine preference over water of UChB rats that had voluntarily consumed morphine for 57 days were morphine deprived for six days and intranasally treated with a single dose of secretome derived from preconditioned MSCs or vehicle (indicated by the arrow) on day 61. Relapse morphine intake and morphine preference upon re-access to the 80 and 90 mg/l morphine solutions over water was significantly reduced in secretome treated rats (80% to 85% ****p < 0.0001, two-tailed Student t test) on the two re-access days (blue bars in **A** and **B**) compared with vehicle treated animals (red bars in **A** and **B**). **C** Daily water intake showing that secretome administration during the morphine deprivation period significantly increased water intake (days 64 and 65) (****p < 0.0001, Two-tailed Student t test) upon morphine re-access (blue bar) compared with vehicle-treated animals (red bar). **D** Total fluid intake and animals body weight of UChB rats intranasally treated with secretome derived from preconditioned MSCs (blue circles) or vehicle (red circles) during the deprivation period. Data shows that these parameters were not affected by secretome administration. Data are presented as mean ± SEM; n = 6 for each experimental condition.

After eight weeks of continuous voluntary oral morphine consumption, animals were deprived of morphine solutions for six days. Following four days of deprivation rats were administered a single intranasal dose of secretome (25μg protein) derived from 1×10^6^ preconditioned MSCs or vehicle while the deprivation was continued for two additional days, after which animals were allowed re-access to the 80 and 90 mg/l morphine solutions for two days (water was always available through all the experiment). The single-dose secretome administration induced a 85% reduction of morphine relapse intake (p < 0.0001, Two-tailed Student t test) assessed 24 hours after morphine re-access, compared to vehicle-treated rats (2.9 ± 0.6 mg morphine/kg/day for secretome-treated animals versus 14.3 ± 0.5 mg morphine/kg/day for vehicle-treated animals), an effect that was maintained in the second re-access day (Fig. 6A). Secretome administration during the deprivation period also reduced morphine preference over water upon re-access (Fig. 6B) and increased water intake (Fig. 6C), without affecting total fluid intake or animal weight (Fig. 6D).

## Discussion

Studies conducted on a model of opioid dependence in Wistar rats which reproduce a severe naloxone-induced withdrawal [10], demonstrated that secretome derived from preconditioned human mesenchymal stem cells administered intranasally and systemically (i) virtually abolished morphine dependence as shown by a > 95% reduction of voluntary morphine consumption, (ii) fully suppressed morphine-induced brain oxidative stress determined by the normalization of increases in GSSG/GSH ratio in the hippocampus and lipid peroxidation in the neostriatum, and (iii) inhibited neuroinflammation evidenced by a full reversal of astrocytic morphological changes induced by chronic morphine self-administration.

The second model of voluntary oral morphine intake was implemented in rats bred as heavy alcohol drinkers (UChB), since these animals have shown a strong drug-permissive genetic background. In such a model, rats orally self-administered morphine solutions of increasing concentrations after systemic morphine administration. In this animal model, intranasal administration of a secretome dose once every five days for 15 days (i) inhibited voluntary morphine intake by 72%, (ii) normalized the GSSG/GSH ratio and the lipid peroxidation index and (iii) reduced neuroinflammation evidenced as a full reversal of astrocyte morphological changes induced by chronic morphine self-administration in the hippocampus, an area related to memory consolidation.

The inhibition of morphine intake reported in both models is consistent with the finding that secretome antagonized both the oxidative stress and the neuroinflammation which characterize the use of addictive drugs [8–10]. Indeed, morphine consumption induces oxidative stress and elevates NO synthesis following μ-opioid receptor activation [8, 11], while neuroinflammation results from the use of opioids [12], mainly activating NFκB signaling leading to the production of pro-inflammatory mediators [10, 13, 14]. The results in the present study are in line with previous reports in animal models of opioid consumption showing that systemic administration of ibudilast, an anti-inflammatory molecule that reduces TNF-α levels, reduces opioid withdrawal [56], while intraperitoneal administration of the antioxidant N-acetyl-cysteine, which improves glutamate uptake, also reduced relapse [57]. In the same line, Motaghinejad et al. reported that the daily intraperitoneal administration of the polyphenol curcumin, with recognized antioxidant and anti-inflammatory properties, lowered the increased levels of brain oxidized glutathione and lipid peroxidation in morphine-treated rats [58], attenuating morphine tolerance and dependence. Thus, the above studies strengthen the idea that blocking the inflammatory and pro-oxidative effects of morphine may provide a promising therapeutic strategy.

Brain oxidative stress and neuroinflammation following the use of addictive drugs [8, 10, 59]; interact in a long-acting vicious cycle which self-perpetuate each other and inhibit the tripartite synaptic glutamate removal by the astrocyte glutamate transporter GLT-1. Indeed, a hyperglutamatergic tonus has been reported for all addictive drugs [60]; in line with the finding that reinstatement of heroin seeking by conditioned cues is associated with glutamate overflow in the nucleus accumbens [61], and with the observation that glutamate and malondialdehyde (MDA) levels and GSSG/GSH ratio were progressively increased in the brain of morphine treated mice [62, 63]. The potent anti-inflammatory and antioxidant effects of MSC-derived secretome are well documented in animal models of stroke [64], perinatal asphyxia [65] and traumatic brain injury [66], where behavioral dysfunctions and markers of neuroinflammation and oxidative stress are reduced after secretome administration.

It is noted that MSC administration has been tested for the treatment of *opioid tolerance*, one of the complications commonly seen after chronic opioid medical use, which is also associated with neuroinflammation, but in the spinal cord [67]. This report indicated that the intravenous administration of rat MSCs to animals that had received daily morphine injections significantly reduced opioid tolerance, restoring the sensitivity to morphine [67]. In line with our results, the reported reduction of morphine tolerance was related to the ability of MSCs to reduce inflammation, decreasing the activation of astrocytes in the spinal cord [67]. Previously, in animal models of ethanol and nicotine dependence, we reported that the intranasal administration of MSC-derived secretome was able to reduce both astrocytic activation and microglial density [34], while for opioid dependence astrocytes appear as the main players in the regulation of opioid relapse.

In the present studies, molecular markers of opioid-induced dependence were examined in prefrontal cortex and nucleus accumbens since these brain areas are highly involved in morphine addiction [68]. As previously reported, we observed that chronic morphine intake induced a significant increase in OPRM1 mRNA levels, the main morphine receptor, in prefrontal cortex and nucleus accumbens [10], but also an unexpected increase in mRNA levels of the glutamate transporters GLT-1 and xCT. The reason for the increase in mRNA levels of glutamate transporters by chronic morphine intake is not known. Nevertheless, these could be associated with a compensatory mechanism related to an increased oxidation of glutamate transporters induced by ROS generated by the chronic morphine consumption, since these transporters present complex regulations [69]. Secretome administration had no impact on morphine-induced mRNA levels of these molecules.

While treatment of opioid-dependent patients without prior waiting for a protracted opioid withdrawal, as shown in the models presented here appears promising, abstinent addicts are at high risk of relapse due to conditioned craving elicited by drug-paired cues [70], leading to a relapse rate of more than 90% after the first month of abstinence [71]. Thus, we also evaluated whether secretome administration given only during the deprivation period could also reduce opioid intake upon re-access. We observed that a single intranasal administration of secretome during the morphine withdrawal period markedly reduced (85%) relapse opioid intake upon re-access, suggesting that MSC-derived secretome could be a useful biodrug for the treatment of opioid addiction.

It is noted that in the Wistar rat (model 1) study, aiming at blocking both the peripheral [72] and central effects of morphine [73], MSCs secretome was administered a single time both intravenously and intranasally. This dual route fully abolished (>95%) morphine dependence intake, an effect observed mostly within the first 48 hours. Such a finding is noteworthy given that in this animal model, naloxone administration generates a most pronounced withdrawal reaction [10]. The UChB rat (model 2) studies showed that by itself the intranasal route was only partially effective in reducing self-administration while a single dose was most effective in reducing post-deprivation relapse (80-85%). The intranasal route may be valuable for the withdrawn opioid user for self-application after a methadone tapering period.

Further studies are required to determine the full duration of the anti-relapse action of secretome and the number of doses needed to maintain the observed therapeutic effects. However, since intranasal administration of MSC-derived secretome has also shown to have potent therapeutic-like effects in animal models of alcohol and nicotine addiction [34], data suggest that this biodrug could have therapeutic potential for polydrug users; a frequent situation [74] for which there is no FDA approved medication [75].

It is noted that opioid substitution has become the treatment of choice for heroin dependence, and methadone and buprenorphine are the substitution drugs most often prescribed. Importantly, polydrug use is particularly prevalent among this group of patients (EMCDDA 2009). In the Netherlands, one half of the methadone users reported cocaine as their secondary drug (IVZ, 2004). Cocaine use can also increase alcohol use, and about 60% of patients in a methadone maintenance program who also used crack cocaine reported using alcohol to come down [76]. In Barcelona, more than one-third of patients were found to be polydrug users when they began methadone maintenance treatment [77]. In essence, polydrug users become opioid dependent, thus greatly adding to the number users at risk of a fentanyl overdose.

A question that can be asked is whether the opioid dependent patient receiving secretome will change to another drug. It is known that the use of several substances by an individual reflects the replacement of one drug by another, due to changes in price, availability, legality, or fashion [78]. Thus, it can also be suggested that drug changes may also occur if there is a secretome-reduced pharmacological potency of the opioid. However, several studies suggest a common mechanism of relapse for different drugs of abuse [17]. Such a view might explain our reports that mesenchymal stem cells and/or its secreted products inhibit drug relapse of several drug types including ethanol, nicotine and now opioids [30, 31, 34, 79, 80].

If the findings in present studies are successfully reproduced in the clinical situation, the administration of mesenchymal stem cell secretome to opioid dependent patients could also have a major economic impact. The Pew-Trust in 2021 [81] estimated the cost of the opioid epidemic In the United States to be $131.8 billion dollars (Health care cost $35 billion; Criminal Justice 14.8 billion; Loss of productivity due to premature death due to overdose and productive hours due to AUD and incarceration: $92 billion).

Overall, the studies conducted show that the administration of secretome derived from human preconditioned MSCs abolishes the opioid-induced neuroinflammation and brain oxidative stress, leading to a significant reduction in chronic opioid intake and opioid relapse. The reported preclinical data may have translational value in reducing the opioid epidemic.

## Supplementary information

Supplementary Figure Legends

Supplementary Figure 1

Supplementary Figure 2

Supplementary Figure 3

Supplementary Table 1
