# Supplementary material for: Effect of human mesenchymal stem cell secretome administration on morphine self-administration and relapse in two animal models of opioid dependence
Source: Transl Psychiatry. 2022 Nov 4;12:462. doi: 10.1038/s41398-022-02225-0 (PMC9636200; doi:10.1038/s41398-022-02225-0)

**Effect of human mesenchymal stem cell secretome administration on morphine self-administration and relapse in two animal models of opioid dependence**

María Elena Quintanilla, PhD^1#^; Mauricio Quezada,PhDc^2#^; Paola Morales, PhD^1,3,4^; Pablo Berríos-Cárcamo, PhD^2^; Daniela Santapau, MS^2^; Marcelo Ezquer, PhD^2^; Mario Herrera-Marschitz, PhD^1^; Yedy Israel, PhD^1,2,4^; Fernando Ezquer, PhD^2,4 *^

**SUPPLEMENTARY FIGURE LEGENDS**

**Supplementary Figure 1: Paradigms of voluntary morphine consumption and relapse in two animal models. (A, Wistar rats)** Immediately after weaning, three-week-old female Wistar rats were offered 150mg/l quinine hydrochloride as the only fluid source. Seven days after forced quinine intake, animals were offered for two weeks a two-bottle choice paradigm in which one bottle contained 150mg/l quinine hydrochloride and the other 150mg/l morphine sulphate. Thereafter, the quinine containing bottle was removed, and animals were offered 150mg/l morphine sulfate and water for two additional weeks. After 22 days of voluntary morphine intake animals were treated with a simultaneous intranasal and intravenous administration of secretome derived from preconditioned MSCs or vehicle. After 30 days of morphine availability animals were morphine deprived for 12 days and treated with two simultaneous intranasal and intravenous administrations of secretome or vehicle (on day 2 and day 9 of the deprivation period) and allowed morphine re-access for 24 hours. **(B, UChB rats)** eight-week-old female UChB rats were administered a daily dose of 40mg/kg i.p. of morphine hydrochloride once a day for 9 consecutive days. On day 10, each cage was fitted with a second drinking bottle containing a solution of morphine with increasing concentrations on successive days (6 to 50mg/l on days 10 to 58). From days 59 to 89 rats were given concurrently free choice access between three bottles one containing water and the other two with 44 and 50mg/l morphine sulfate solutions. After 10 weeks of continuous voluntary morphine consumption, animals received three intranasal doses of secretome or vehicle in a period of two weeks (days 75, 80 and 85). To evaluate morphine post-deprivation relapse, four days after the last intranasal dose of secretome animals were deprived of morphine solutions for five days and were treated with a fourth dose of secretome or vehicle during the first day of deprivation. Subsequently, rats were allowed re-access to the 44- and 50mg/l morphine solutions for one day. **(C, UChB rats)** eight-week-old female UChB rats were administered a daily dose of 40mg/kg i.p. of morphine hydrochloride once a day for 11 consecutive days. On day 12, each cage was fitted with a second drinking bottle containing a solution of morphine with increasing concentration on successive days (6 to 90mg/l, days 12 to 42). From days 43 to 57 rats were given free choice access between three bottles one containing water and the other two with 80 and 90mg/l morphine sulfate solutions. On day 58 animals were deprived of the morphine solutions for six days and were treated with a single intranasal dose of secretome or vehicle during the fourth day of deprivation. Rats continued under morphine deprivation for two additional days and thereafter animals were allowed re-access to the 80 and 90mg/l morphine solutions for two days.

**Supplementary Figure 2: Intranasal and intravenous administration of secretome derived from human preconditioned MSCs to rats that had chronically consumed morphine did not modify total fluid intake or body weight. (A)** Total fluid intake and **(B)** animals body weights of Wistar rats that after been voluntarily consuming morphine for 22 days were intravenously and intranasally treated with secretome derived from preconditioned MSCs (blue circles) or vehicle (red circles). Data showed that these parameters were not affected by secretome administration, indicating that therapeutic effects induced by this treatment were specific for morphine intake. Data are presented as mean ± SEM; n=9 for each experimental condition.

**Supplementary Figure 3: UChB rats receiving intraperitoneal daily morphine administrations and subsequently allowed two-bottle access to water and to morphine solutions of increasing concentrations displayed gradual increases of morphine intake. (A)** Naïve females UChB rats were intraperitoneally injected with a daily dose of morphine (40mg/kg) for a 9-day period followed by a free-choice access between water and progressive escalating morphine concentrations (6mg/l to 50mg/l). Following the intake of the 44 and 50mg/l morphine solutions rats voluntarily consumed a constant amount of 7.7 ± 0.3mg of morphine/kg/day (mean ± SEM). See also Supplementary Figure 1B. One-way ANOVA of all voluntary oral morphine intake data indicated a significant effect of the concentration of the morphine solution offered to the rats [*F_(morphine concentration 7,56)_*= 310.5, *p* < 0.0001]. Tuckey`s post-hoc test indicates that every increase in the concentration of morphine in the range of 6mg/l to 50mg/l resulted in a significant increase (p<0.0001) in daily morphine intake expressed as mg morphine ingested/kg body weight (n=8). **(B)** UChB rats were offered higher concentrations of morphine over those indicated above. Naïve females UChB rats were intraperitoneally injected with a daily dose of morphine (40mg/kg) for a 11-day period followed by a free-choice access between water and progressive escalating morphine concentrations (6mg/l to 90mg/l). Following the intake of the 80 and 90mg/l morphine solutions rats voluntarily consumed a constant amount of 14.2 ± 0.3mg of morphine/kg/day (mean ± SEM). See also Supplementary Figure 1C. One-way ANOVA of all voluntary oral morphine intake data indicated a significant effect of the concentration of the morphine solution offered to the rats (p< 0.0001). Tuckey post-hoc test reveals that every increase in the concentration of morphine in the range of 6mg/l to 90mg/l resulted in a significant increase (p<0.0001) in daily morphine intake expressed as mg morphine ingested/kg body weight (n=12).

**Supplementary Table 1: Specific primers for RT-qPCR amplifications.**

**Supplementary Figure 1**


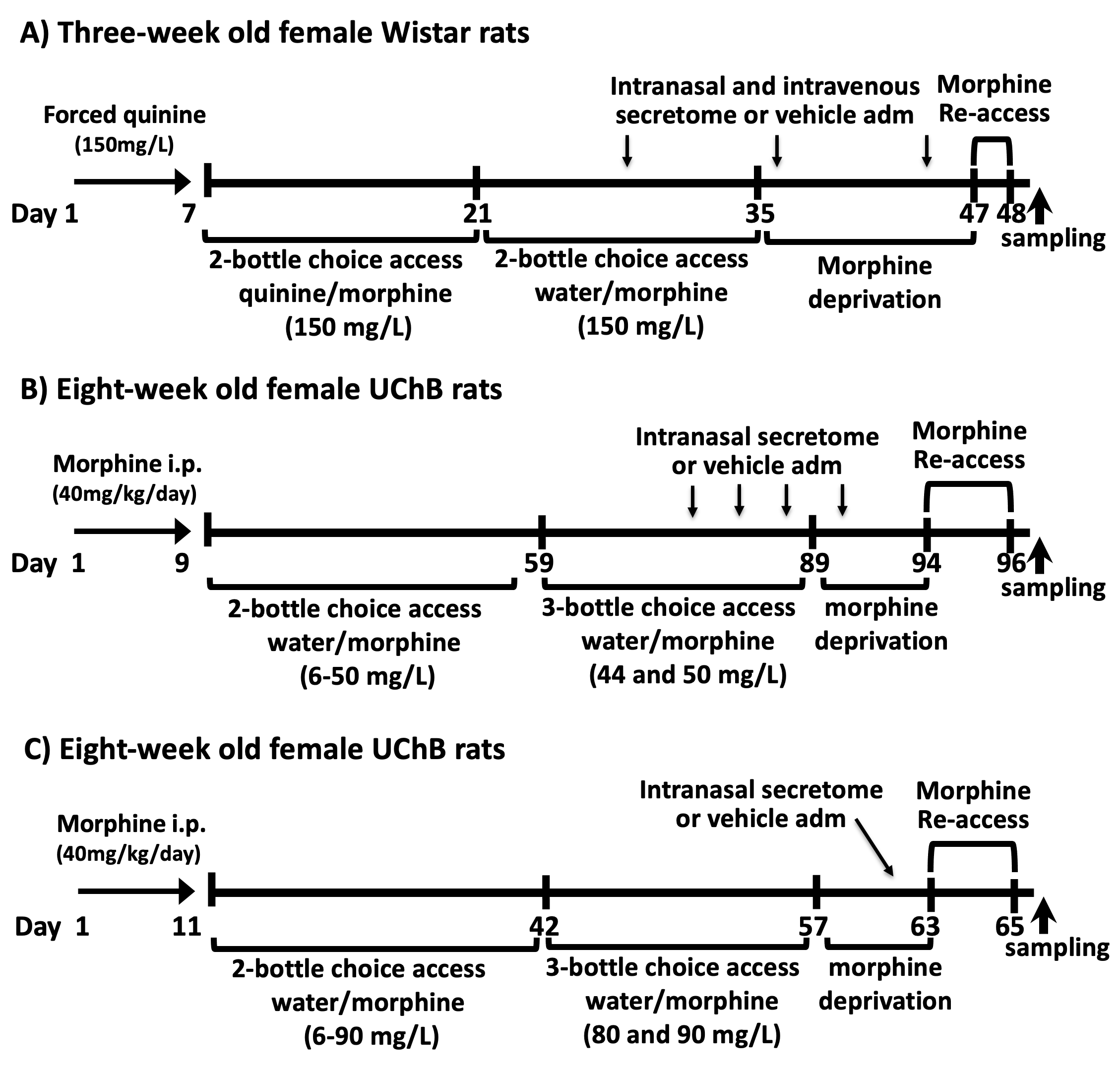


Supplementary Figure 2


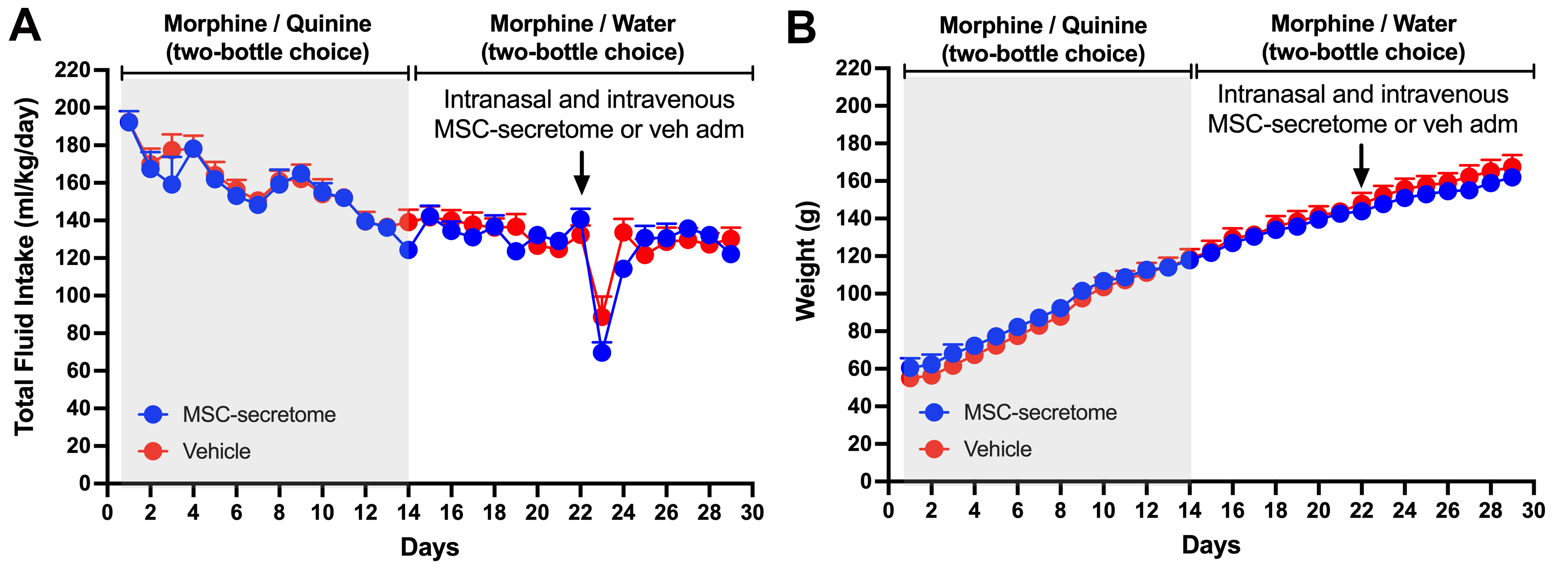


Supplementary Figure 3


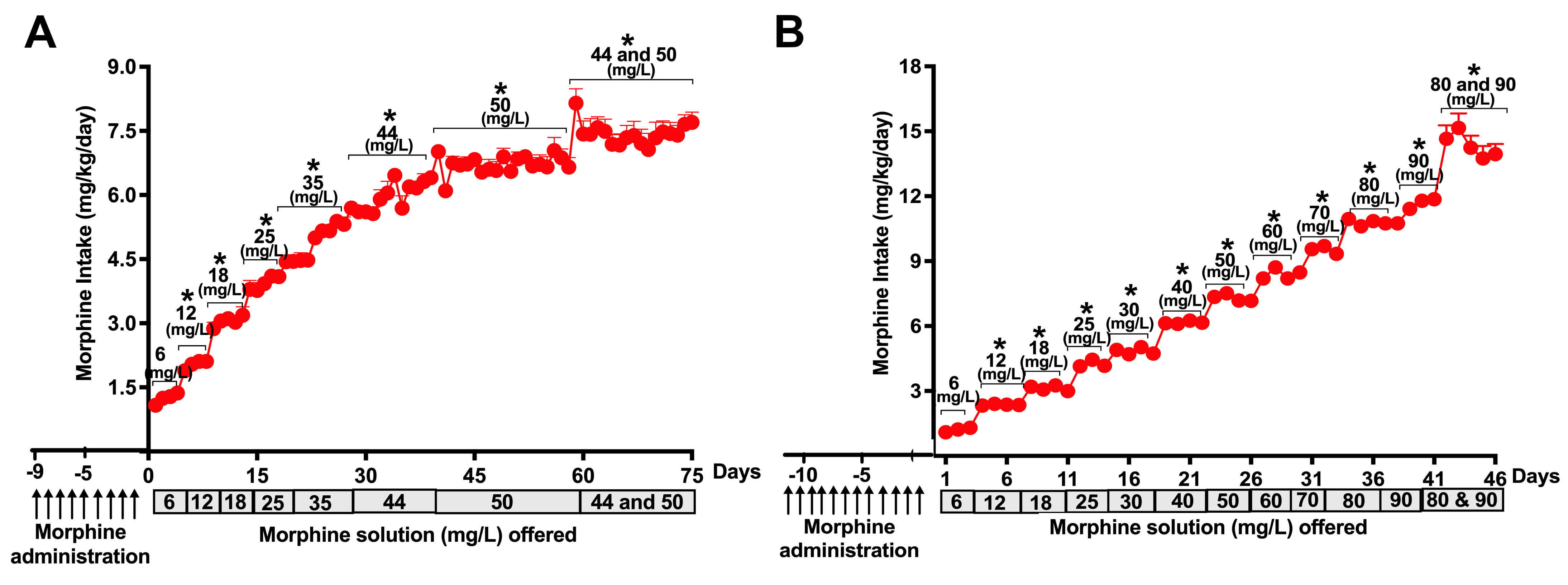


Supplementary Table 1


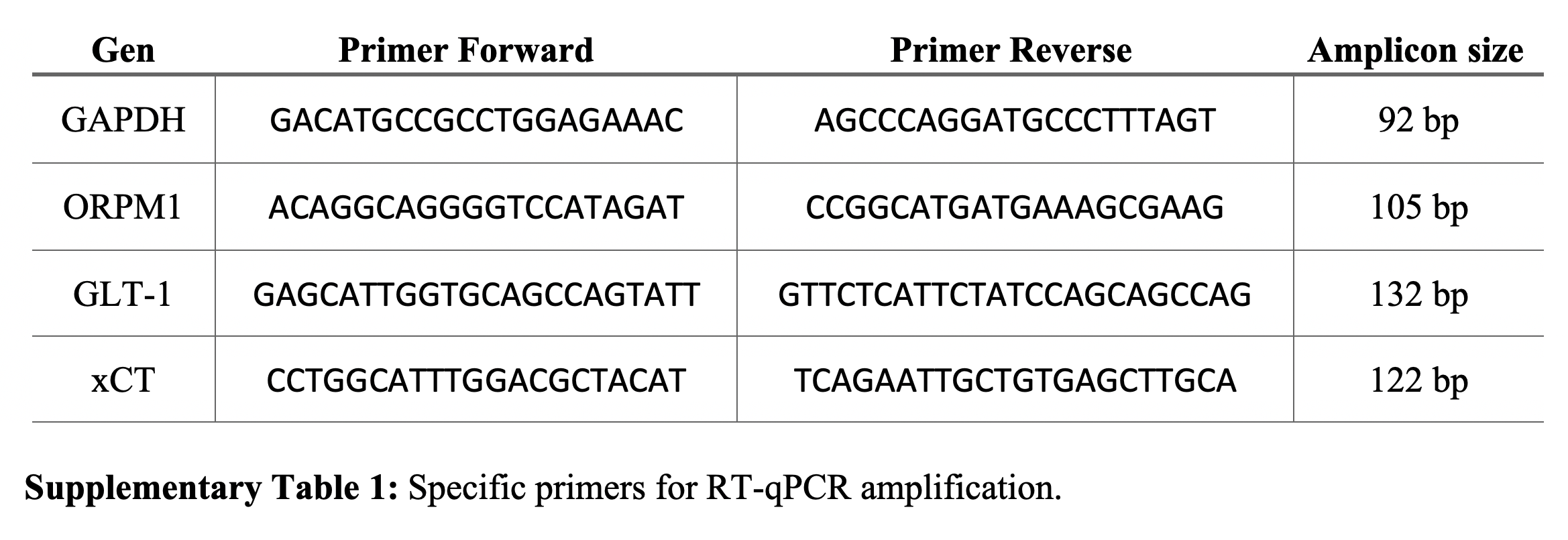

Supplement: Supplementary file 1 — Supplementary Figure Legends [file 41398_2022_2225_MOESM1_ESM.docx]
