# Supplementary figures and images for: Effect of human mesenchymal stem cell secretome administration on morphine self-administration and relapse in two animal models of opioid dependence
Source: Transl Psychiatry. 2022 Nov 4;12:462. doi: 10.1038/s41398-022-02225-0 (PMC9636200; doi:10.1038/s41398-022-02225-0)

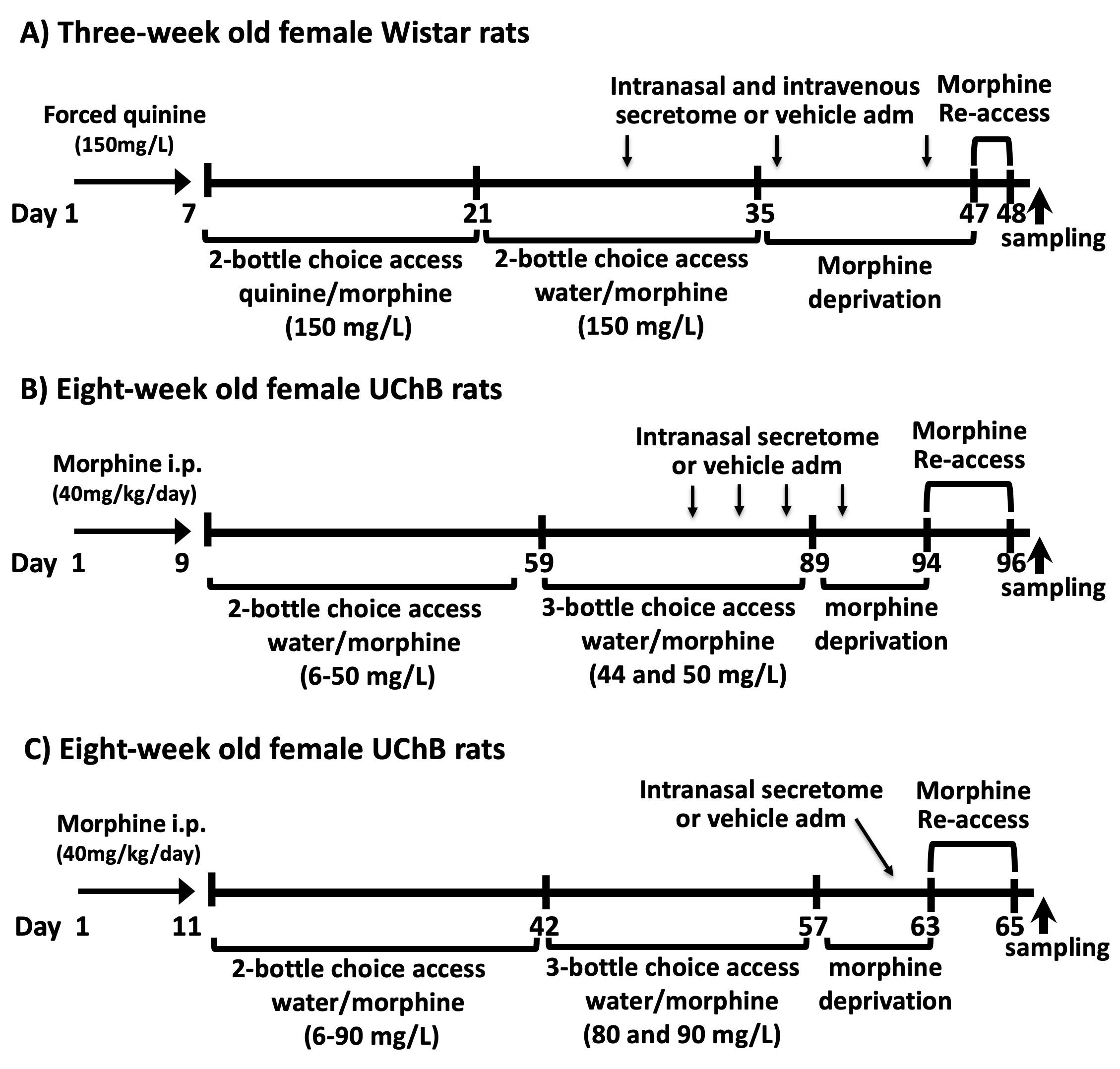

Supplement: Supplementary file 2 — Supplementary Figure 1 [file 41398_2022_2225_MOESM2_ESM.tif]

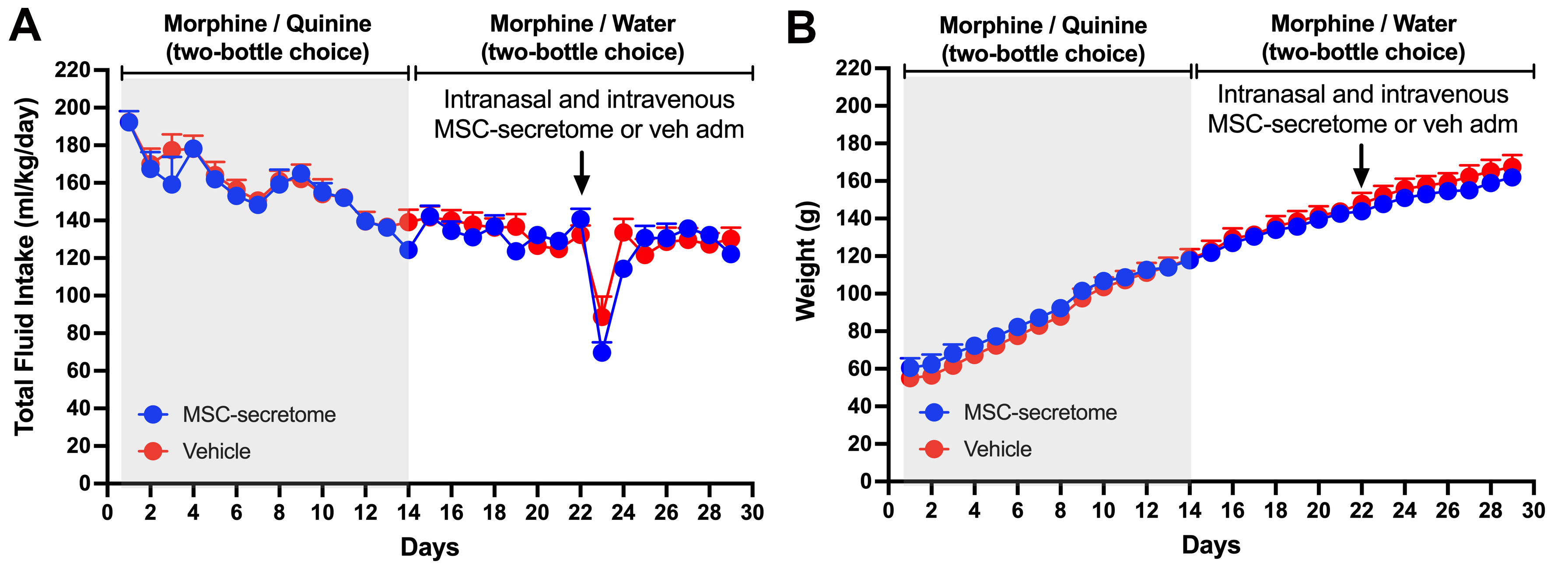

Supplement: Supplementary file 3 — Supplementary Figure 2 [file 41398_2022_2225_MOESM3_ESM.tif]

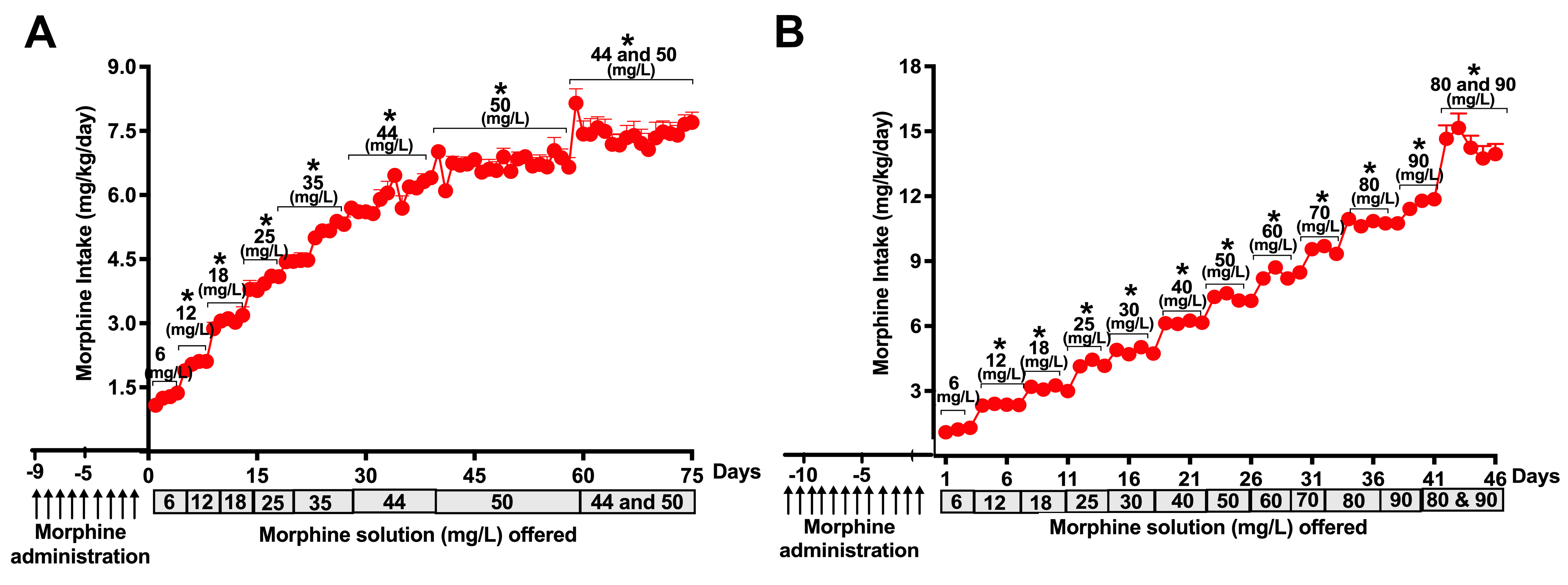

Supplement: Supplementary file 4 — Supplementary Figure 3 [file 41398_2022_2225_MOESM4_ESM.tif]

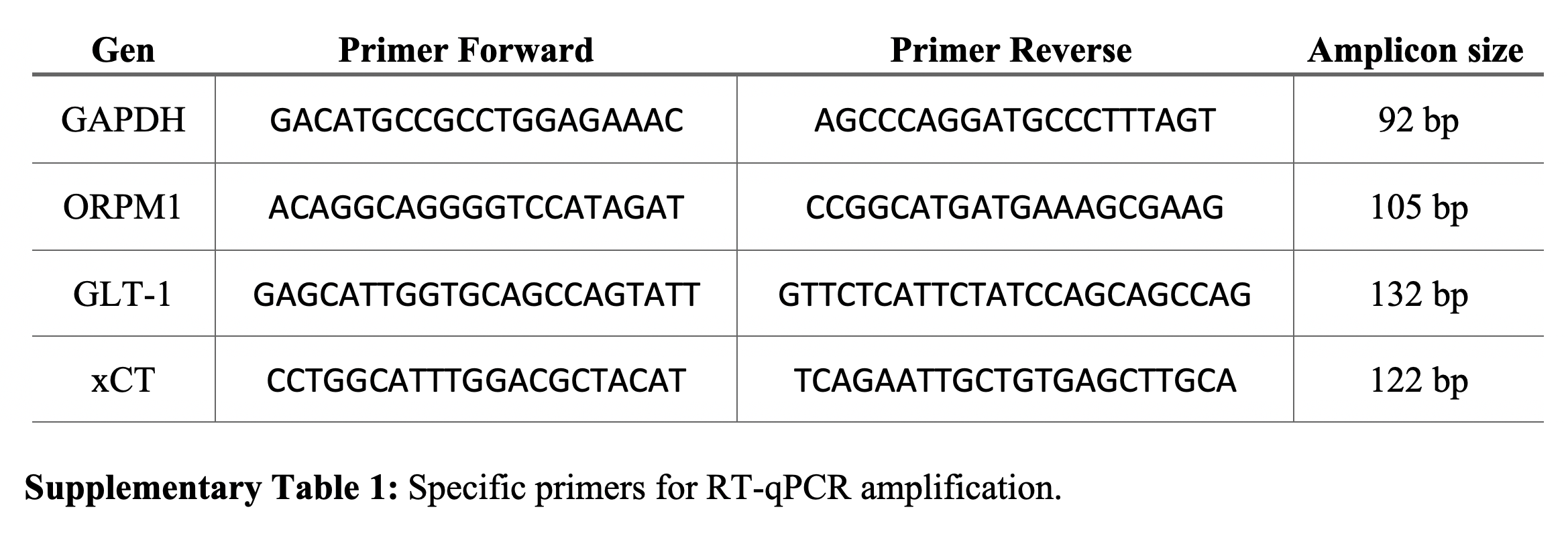

Supplement: Supplementary file 5 — Supplementary Table 1 [file 41398_2022_2225_MOESM5_ESM.tif]
